# Supplementary material for: Transient Global Amnesia (TGA): Is It Really Benign? A Pilot Study on Blood Biomarkers
Source: Int J Mol Sci. 2025 Mar 14;26(6):2629. doi: 10.3390/ijms26062629 (PMC11941937; doi:10.3390/ijms26062629)
Supplement: Supplementary file 1 [file ijms-26-02629-s001.zip › ijms-3499062-supplementary.pdf]

Supplementary Materials

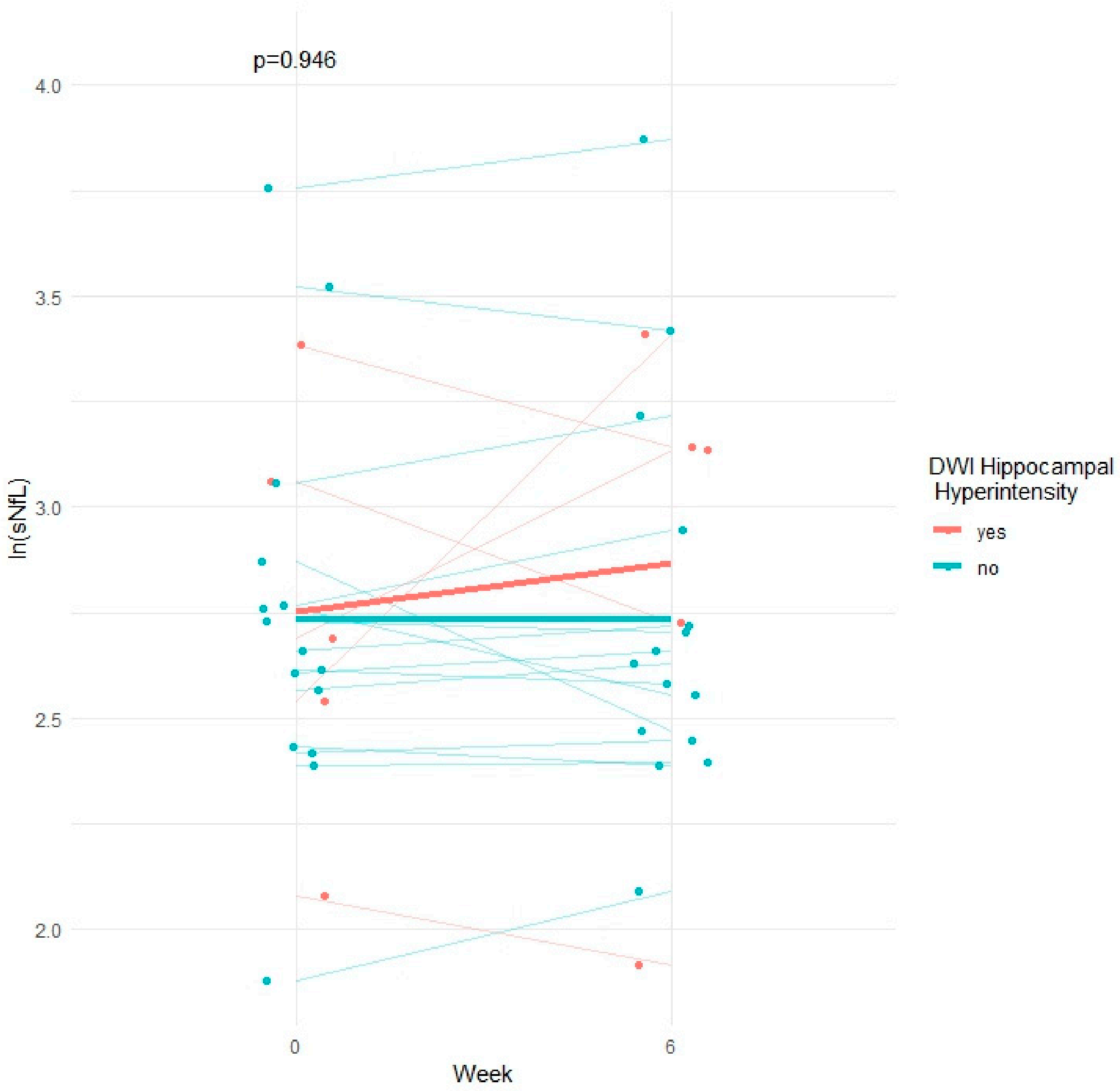

Supplemental Figure S1. Log-transformed serum neurofilament light chain (sNfL) concentrations (pg/mL) measured within 24 to 48 hours after symptom onset (t0) and at six weeks (t1) in 20 patients diagnosed with TGA, stratified by the presence or absence of diffusion-weighted imaging (DWI)-detected hippocampal lesions.

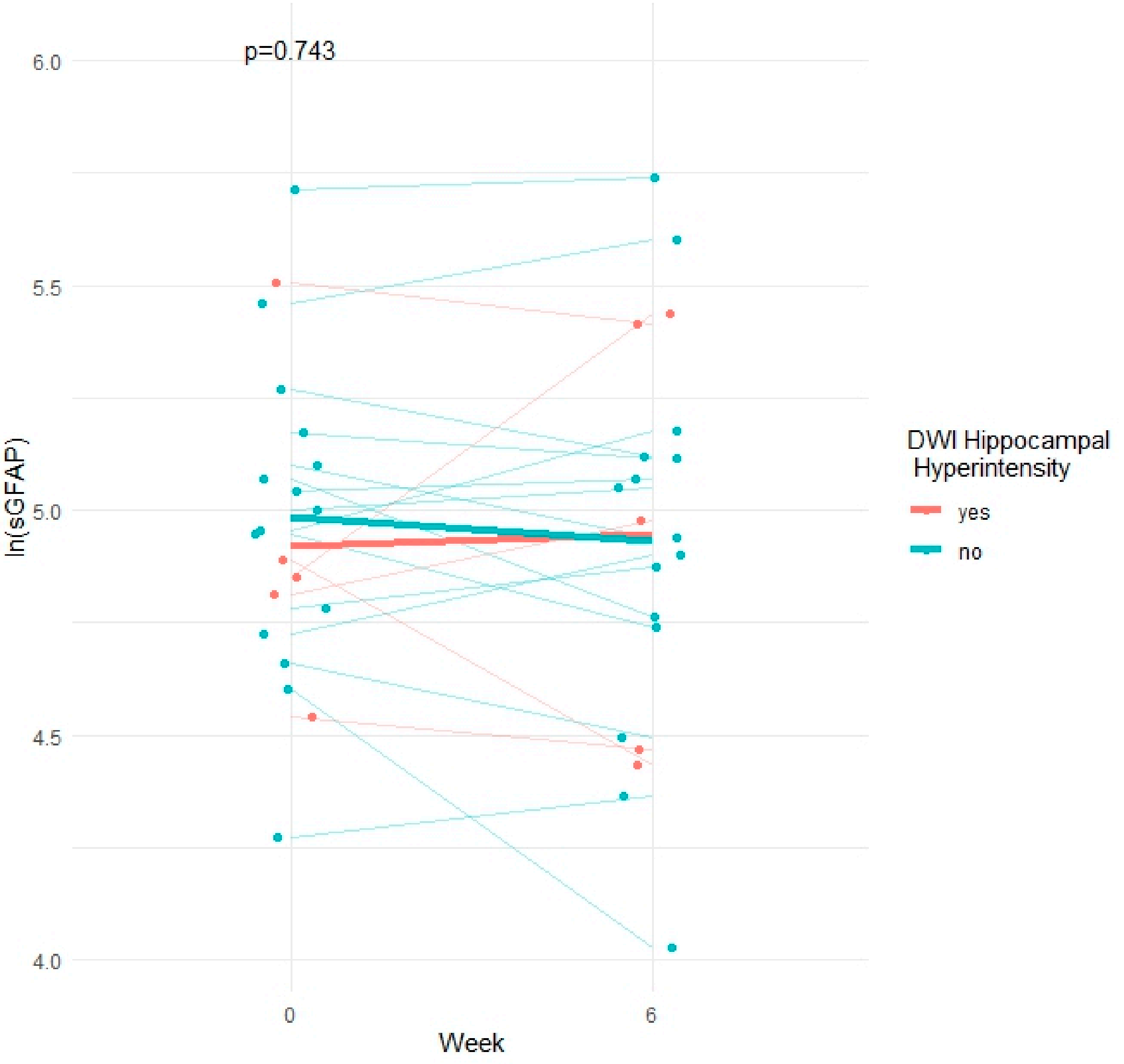

Supplemental Figure S2 Log-transformed serum glial fibrillary acidic protein (sGFAP, in pg/mL) values measured within 24 to 48 hours of symptom onset (t0) and six weeks after onset (t1) in 20 patients with TGA, grouped by the presence or absence of diffusion-weighted imaging hippocampal lesions.

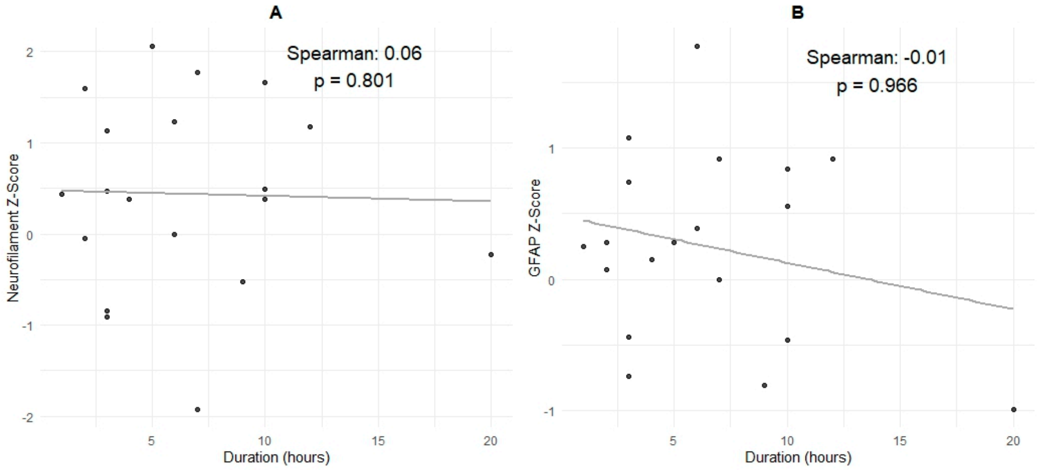

Supplemental Figure S3. Scatter plots depicting the correlation between the duration of TGA (in hours) and biomarker levels as measured on admission (t0). (A) Relationship between duration and neurofilament light chain (sNfL) Z-score (Spearman's ϱ = 0.06, p = 0.801). (B) Relationship between duration and glial fibrillary acidic protein (sGFAP) Z-score (Spearman's ϱ = -0.01, p = 0.966). Gray regression lines represent linear fits for visualization purposes. No significant associations were observed.
